# Salivary Lymphocyte Phenotypes Differ from Blood and Serve as a Model for Other Mucosal Fluids

**DOI:** 10.33696/immunology.7.233

**Published:** 2025

**Authors:** Muruganantham Lillimary Eniya, Shervin Dokht Sadeghi Nasab, Albert Judith, Frederick Clasen, David Moyes, Saeed Shoaie, Newell Johnson, Priya Kannian, Stephen Challacombe

**Affiliations:** 1 The Voluntary Health Services, Chennai, India; 2 Faculty of Dentistry, Oral & Craniofacial Sciences, King’s College London, UK; 3 Griffith University Dental School, Queensland, Australia

**Keywords:** Lymphocyte phenotyping, Stimulated whole mouth fluid, Peripheral blood, Flow cytometry, Saliva, Mucosal fluids

## Abstract

**Background & Objectives:**

The oral cavity is part of the mucosal immune system of the body, embracing all mucosae including lungs, gut and nose. The oral mucosa is the gateway for a plethora of gastrointestinal and respiratory antigens and is capable of mounting a very strong mucosal immune response. Mucosal immunity is structurally and functionally similar in all mucosae and, soluble mediators including cytokines, chemokines, immunoglobulins and other proteins have been well studied in many diseases. However, the roles of mucosal immune cell phenotypes remain less studied causing setbacks in our understanding of the disease pathogeneses.

**Methods:**

We have reviewed the importance of immune phenotyping of the cells in mucosal secretions and the employment of flow cytometry as a reliable tool for this purpose. We previously showed that CD3, CD4, CD8, Th1, and Th2 cells can be detected in stimulated whole mouth fluid (SWMF) in a reproducible and consistent manner, and their ratios may differ from those in the peripheral circulation. We now show for the first time that other salivary lymphocytes Th17, Th22, Tfh, Tregs, NKT, NK, ILC1, ILC2, and ILC3 cells can also be detected and quantified using flow cytometry. PBMC and SWMF from 119 participants were tested by flow cytometry.

**Results:**

Mean frequencies of all the immune cells were detected in a reproducible and consistent manner. In the SWMF the mean frequencies of Th17, Th22, Tfh and NK cells were greater than PBMC. These phenotypes in SWMF showed a negative correlation with PBMC suggesting mucosal origin and specificity.

**Conclusions:**

Our findings strongly suggest that flow cytometry can be employed to detect a wide range of lymphocyte phenotypes in SWMF, a hypotonic secretion. Due to the similarities among the various mucosal secretions, this technique could be explored as a promising tool for understanding immunopathogenesis of infectious and non-infectious diseases using mucosal secretions.

## Commentary

The oral cavity is lined by the oral mucosal membrane, which provides integrity to the internal environment via the mucosal immune system [1]. The oral mucosa consists of induction sites and effector sites [2]. The epithelial layer and the Lamina Propria (LP) comprise the effector sites. The epithelial cells, Langerhans cells, monocytes, macrophages, and dendritic cells in these effector sites encounter the large repertoire of antigens (including food, allergens, and infectious agents) that enter the oral cavity. They process the antigens and then migrate to the induction sites carrying the foreign antigens that need to be eliminated and present them to the lymphocytes. Induction sites include lymphoid follicles, draining lymph nodes of salivary glands, Waldeyer’s ring (primarily the tonsils and adenoids), duct associated lymphoid tissue (DALT) of hundreds of minor salivary glands which together are part of the mucosa-associated lymphoid tissue (MALT). The lymphocytes in these sites are primed by the antigen presenting cells and thus adaptive immunity is elicited [3].

The oral mucosa is continuously bathed by saliva secreted by the major and minor salivary glands. In dentate subjects, there is a constant passage of serum and its by-products into the oral cavity through the crevicular spaces in the pockets of each tooth [2]. Circulating antibodies get into the oral cavity during this process [4]. Local secretory IgA (SIgA) antibodies and other cytokines/chemokines are also produced by the cells in the effector and induction sites that orchestrate the elimination of the invading pathogens. Thus, saliva is an excellent non-invasive sample for diagnostics as well as research to study the mucosal immune response in the oral cavity. Mortazavi *et al*. have concluded that the stimulated whole mouth fluid (SWMF) provides remarkable outcomes with regards to the total volume of saliva, salivary flow rates, pH, total protein concentration, and DNA quality and concentration [5]. Saliva poses certain limitations due to its hypotonicity and constant turn over due to the swallowing process. Soluble mediators including IgA/SIgA, cytokines/chemokines, biochemical mediators like cortisol, electrolytes, and proteins have been studied extensively. However, little progress has been made in optimizing a technique for the detection of live cells in the SWMF or several other mucosal fluids.

Determination of the immune phenotypes in the SWMF would also provide great opportunities to understand the mucosal immune responses in many diseases with both infectious and non-infectious causes. Immunophenotyping by flow cytometry appears to be a robust tool to delineate the cellular mediators in the body fluids. Vidovic *et al*. had initially shown that the neutrophils, monocytes, and CD3/CD4 T cells in the saliva can be detected by flow cytometry [6]. Epithelial cells are the most abundant cell type followed by neutrophils, monocytes and lymphocytes, in the order of abundance [6]. Subsequently we have shown that the five major T cell phenotypes – CD3, CD4, CD8, Th1, and Th2 cells can be detected in a technically reproducible (by testing split sample duplicates) and biologically consistent (by testing biological replicates collected four weeks apart) manner in SWMF samples using flow cytometry [7].

The CD3, CD4, and CD8 T cell frequencies reported in our study corroborated with other studies using the same technique [8–10]. We showed significant correlations between the percentages of CD3, CD4, Th1, and Th2 cells in peripheral circulation and SWMF suggesting a strong link between the induction of the immune cells in the local milieu and the peripheral circulation. Therefore, there is value to extend these reproducibility and consistency studies to other common leucocytes (Th17, Th22, Tregs, Tfh, ILC1, ILC2, ILC3, NK, and NKT cells) in the SWMF and to other mucosal fluids in order to determine any local mucosal contributions. Studies of other mucosal fluids include nasal and bronchial lavage samples [14] and cervico-vaginal secretions [15,16] where flow cytometry has been used to detect and analyze a limited spectrum of immune cells.

Blood and SWMF samples were processed as described by us previously [7]. Briefly, PBMC were isolated from whole blood using Ficol gradient (HiMedia, India or Sigma Aldrich, USA). SWMF samples were washed and sedimented by centrifugation. Fifty microliters of both samples were used with anti-human mouse antibodies (BD Biosciences, USA) for flow cytometry (AF488 anti-ROR-γt: clone Q21-559; BV421 anti-CCR4: clone 1G1; PE-Cy7 anti- CCR6: clone 11A9; BB515 anti-CCR10: clone 1B5; AF647 anti-CXCR5: clone RF8B2; BV605 anti-PD-1: clone EH12.1; BV711 anti-ICOS: clone DX29; AF647 anti-Foxp3: clone 259D/C7; BV786 anti-CD25: clone M-A251; BV605 anti-CD56: clone NCAM16.2). The antibody clone details for CD3, CD4, CD8, Th1, and Th2 cells are described previously [7]. Cells were finally resuspended in cold Dulbecco’s PBS and acquired in BD FACSLyric or BD FACS Canto II (BD Biosciences, USA). The relative frequencies of the cell phenotypes were analyzed using BD FACS Suite or FlowJo softwares.

**Figure 1:**
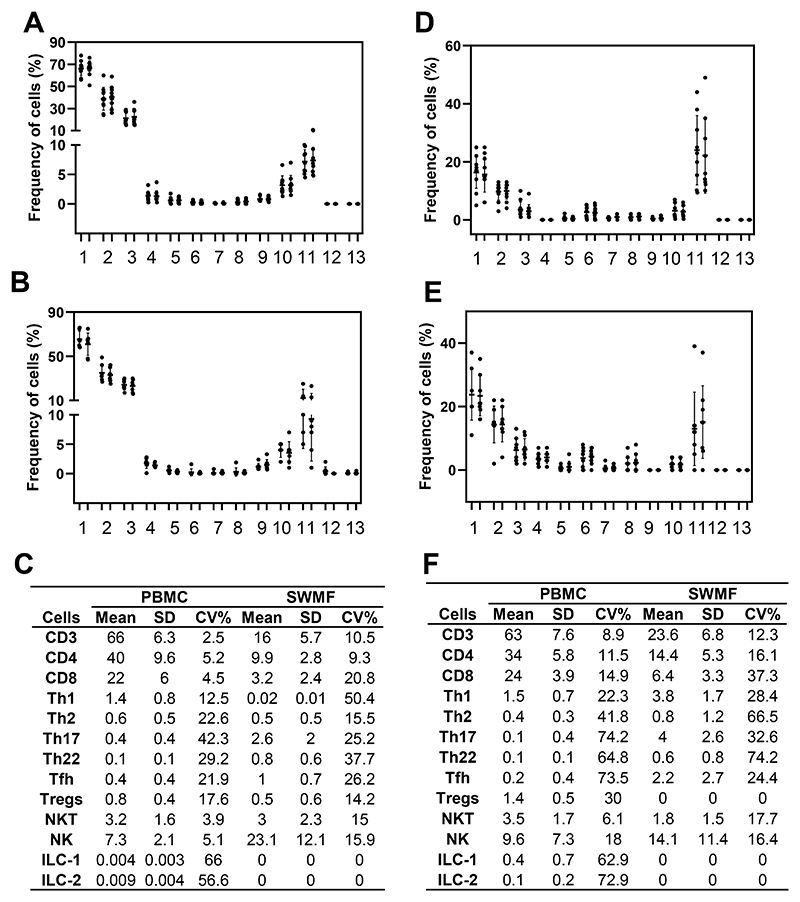
Technical reproducibility and biological consistency in the detection of immune cell types in SWMF compared to PBMC by flow cytometry. X-axis indicates the various immune cell types. Y-axis indicates the frequencies of the cells in percentages. The circles denote each sample and the error bars denote the mean and standard deviations (SD) within each group. **A. Technical reproducibility in PBMC. B. Technical reproducibility in SWMF. C. Table inset** showing the mean, SD and mean of the CV% for the experimental replicates (N=5-11). **D. Biological consistency in PBMC. E. Biological consistency in SWMF. F. Table inset** showing the mean, SD and mean of the CV% for the longitudinal samples collected four weeks apart (N=8). **1** – CD3 T cells, **2** – CD4 T cells, **3** – CD8 T cells, **4** – Th1 cells, **5** – Th2 cells, **6** – Th17 cells, **7** – Th22 cells, **8** – Tfh cells, **9** – Tregs, **10** – NKT cells, **11** – NK cells, **12** – ILC1, **13** – ILC2.

Figure 1 shows the technical reproducibility and biological consistency in the mean frequencies of the various cell types in the PBMC and SWMF. For the technical reproducibility determination, two split sample replicates from healthy controls (HC) were processed (N=5–11) independently based on the adequacy of the samples. For the biological consistency determination, PBMC and SWMF samples were collected four weeks apart from HC. ILC1 and ILC2 cells were not detected in these SWMF samples. ILC3 were not detected in either PBMC or SWMF. The frequencies of the duplicates of each of the cell types are shown in Figure 1A (PBMC) and Figure 1B (SWMF). The frequencies of the cell types in the longitudinal samples are shown in Figure 1D (PBMC) and Figure 1E (SWMF). Mean frequencies and the means of the CV% are shown as table insets (Figure 1C and 1F).

The mean frequencies (calculated over mononuclear leucocytes; Figure 1C) were higher in PBMC than SWMF for CD3 (66% vs 16%), CD4 (40% vs 9.9%), CD8 (22% vs 3.2%), Th1 (1.4% vs 0.02%), Th2 (0.6% vs 0.5%), ILC1 (0.004% vs 0%) and ILC2 (0.009% vs 0%) cell types. For Th17 (0.4% vs 2.6%), Th22 (0.1% vs 0.8%), Tfh (0.4% vs 1.0%) and NK (7.3% vs 23.1%) cells, the mean frequencies were higher in SWMF than PBMC suggesting a more prominent mucosal role for these cell types. Our distribution of the mononuclear cells in SWMF is very different from that of PBMC and similar to that previously shown by Vidovic *et al*. [6].

As shown in our previous study, the mean CV% are usually higher in both sample types when the frequencies are lower. For example: CD3 T cells had a mean frequency of 66% and a mean CV of 2.5%, while ILC1 had a mean frequency of 0.004% and a mean CV of 66% (Figure 1C). High variations are also because of the low probability of proportionally equal division of rare population of cells in a given sample.

Variation is lower in the technical reproducibility experiments for all the cell types than the biological consistency experiments. For example: CD3 T cells in PBMC had a mean CV of 2.5% between split sample duplicates (Figure 1C), but a mean CV of 8.9% between the two biological replicates collected four weeks apart (Figure 1F). The low variation in reproducibility of detecting a particular cell phenotype indicates the robustness and reliability of flow cytometry as a technique to detect the immune cell types.

Biological consistency of cell types is additionally governed by the variation in the presence of these cell types in the peripheral circulation and/or the mucosal secretion at different time points under normal physiological conditions. Some cell types like the NK cells are known to be constantly shuttling in and out of the mucosal compartment. The half-life of most of the immune cells in the resting state are very short ranging between a few hours and a few days [14–17]. SWMF gets constantly replenished due to the normal swallowing action. The distribution frequency of these cells in SWMF at different time points is impacted by many physiological factors, yet the experiments reveal an individual biological consistency over the four-week period.

**Figure 2:**
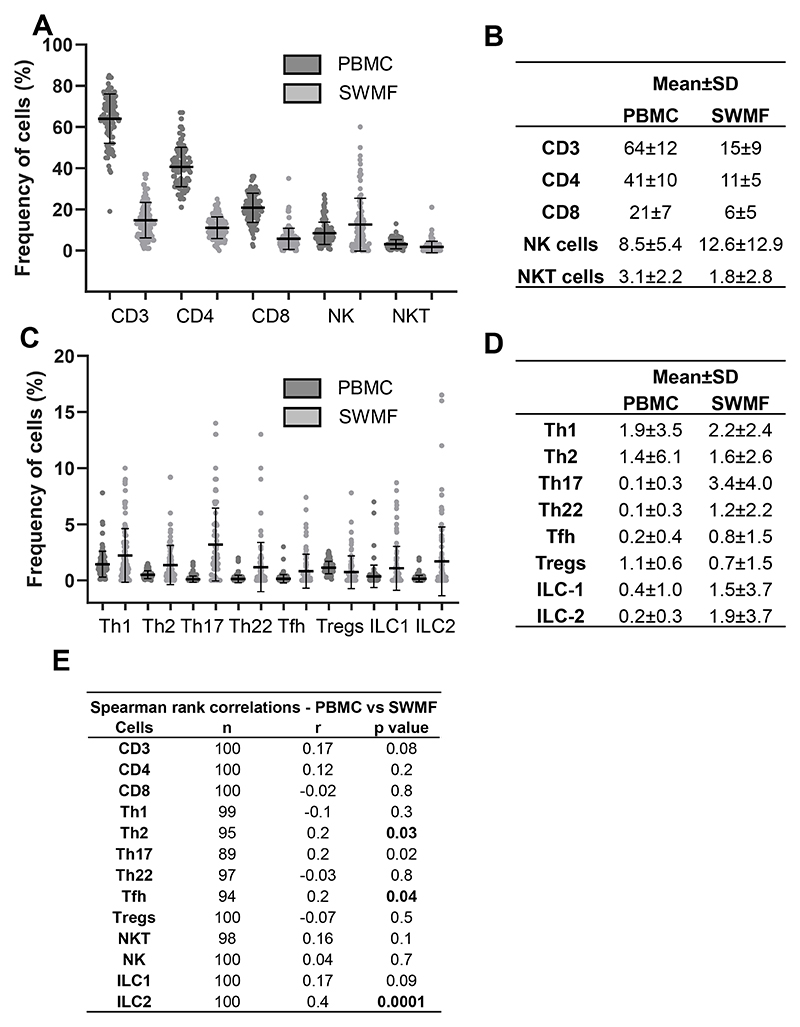
Detectability of the various immune cells in SWMF in comparison to PBMC (N=100). X-axis indicates the various immune cell types. Y-axis indicates the frequencies of the cells in percentages. The black circles denote the cell frequencies in PBMC, the grey circles denote the cell frequencies in SWMF and the error bars denote the mean and SD within each group. **A.** Cell types with higher frequencies. **B.** Table inset showing the mean and SD in PBMC and SWMF for the cell types shown in A. **C.** Cell types with lower frequencies. **D.** Table inset showing the mean and SD in PBMC and SWMF for the cell types shown in C. **E.** Spearman rank correlations between PBMC and SWMF for the various immune cell types. N: Number of Samples, r: Correlation Co-efficient.

Since the initial technical reproducibility and biological consistency experiments were performed with a small sample size, we analyzed these immune cell types in PBMC and SWMF of 100 participants (subset of another study; Figure 2). All the cell types except ILC3 were detectable in both fluids. Cells with higher frequencies are shown in Figure 2A, while those with lower frequencies are shown in Figure 2C. The table insets (Figure 2B and 2D) show the mean ± SD frequencies of the various cell types in both PBMC and SWMF. The mean frequencies were lower in SWMF compared with PBMC for CD3, CD4, CD8, and NKT cells, while the mucosal secretion had higher frequencies for NK, Th17, Th22, Tfh, ILC1 and ILC2 cells. Spearman rank correlation analysis showed positive correlations between the PBMC and SWMF for CD3, CD4, Th2 (p=0.03), Th17, Tfh (p=0.04), NKT, NK, ILC1, and ILC2 (p=0.0001) cells (Figure 2E). Negative correlations were seen for CD8, Th1, Th22, and Treg cells.

It is notable that certain immune cells – Th1, Th2, ILC1, and ILC2 were detected at higher levels in this cohort when compared to the HC group shown in Figure 1. The immunophenotyping results of these 100 participants are discussed here to show the robustness of flow cytometry as a technique to detect the immune cells in SWMF. These 100 participants had recovered from either asymptomatic or mild COVID-19. This could be one of the reasons for some of the higher immune cell frequencies compared to the HC group in Figure 1. The complete data analysis of the immune phenotypes in this cohort will be presented in a forthcoming publication.

Although we have shown that over ten lymphocyte phenotypes can be detected in the oral fluid, a hypotonic mucosal secretion, there are other potentially important cells still to be examined such as mucosa associated invariant T cells (MAIT cells). The latter are found within mucosae and have been implicated in vaccine induced B cell responses [18] and IL-22 production [19], but have not to our knowledge been determined in human mucosal secretions.

Thus, our study demonstrates flow cytometry to be a reliable and robust tool for the detection of a wide variety of immune cell types in the mucosal secretion, which can be compared with that of PBMC. This technique may be used to explore the immune cell phenotypes in oral fluid to understand their role in mucosal immunity at the oral cavity, which is the portal of entry for many respiratory and gastrointestinal pathogens. The technique could also be standardized as a promising tool in other mucosal secretions to understand the role of immune cells in many non-infectious diseases and other infections including SARS-CoV-2 [20].
